# New Complexities and Approaches to Global Health Diplomacy: View from the U.S. Department of State

**DOI:** 10.1371/journal.pmed.1000276

**Published:** 2010-05-11

**Authors:** Kerri-Ann Jones

**Affiliations:** United States Department of State, Washington, D.C., United States of America

## Abstract

As part of the *PLoS Medicine* series on Global Health Diplomacy, Kerri-Ann Jones provides a perspective from the Inited States Department of State on current complexities and challenges in global health diplomacy.

*This article is part of the* PLoS Medicine *Global Health Diplomacy series.*

## Overview

As the world has grown smaller with intensive transportation and communication advances, it has also grown more interdependent. Products and people are constantly on the move and fragile ecosystems are increasingly disturbed as development pushes into new areas. Environmental pressures or ineffective health systems are recognized for their local impact but also seen as potential sources of national and international political, economic, and social tension. In the 21st century it has become essential that foreign policy moves well beyond its earlier, more narrow focus on political, military, and commercial affairs. The urgent global challenges we face—climate change, global health, and food security—are now central to foreign policy and national security concerns.

The term “global health diplomacy” embraces the challenges we face. International diplomatic engagement is essential for building global policies to address health issues, including HIV/AIDS, the current H1N1 pandemic, ongoing pandemic preparedness, insufficient global influenza vaccine supply and production capacity, growing concern over the spread of polio, maternal and child health, and structural health system challenges facing the developing world. Advancing foreign policy and national security objectives is dependent on diplomacy and development.

## HIV/AIDS: A Turning Point

The emergence of HIV was a turning point in the world of international health. That deadly virus brought the world a new message—global health was not only the concern of health and development organizations. When HIV was discovered in the early 1980s, the health research and public health communities were on the front lines of the response. The priorities were to prevent new infections through public health measures while working to develop effective treatments.

In 1995, the US Department of State issued its first US international strategy on HIV/AIDS. It provided guidance to the US diplomatic community around the world, and it formed the basis of numerous US governmental positions taken in multilateral arenas. It focused attention on the lower status of women and girls in many nations, which increases their risk for HIV infection. It also focused attention on losses of highly trained cadres of experts in poorer nations, including teachers and military leaders. Critically, it focused attention on the need for a broad-based approach to include every sector of society to understand how the pandemic was spreading on national and international levels and on how to reduce and prevent the spread of the disease.

With the increasing understanding of the vast toll that HIV/AIDS would take on economies in countries hardest hit, mainly those in Africa, and with military and security concerns rising, the US diplomatic community became engaged. Working with scientists, health professionals, business and military groups, and nongovernmental organizations, the US government recognized the broad societal impacts of AIDS, and that these impacts had moved beyond the health sector. To tackle the challenge, the US response therefore needed to be broad.

That response culminated in the President's Emergency Plan for AIDS Relief (PEPFAR), launched by President George W. Bush in 2003. This response represented the largest single commitment of the U.S. to any specific disease globally, and set out to achieve what many thought impossible. With an initial strategy focused on specific goals in 15 countries, PEPFAR saved millions of lives and helped to create, support, and strengthen health systems in the developing world. Through PEPFAR, the U.S. has directly supported life-saving antiretroviral medicine for more than 2.4 million people, care for nearly 11 million, and prevention of almost 350,000 cases of mother-to-child transmission of HIV.

In addition to these bilateral efforts, the U.S. has strengthened its resolve to support global health through diplomatic and development efforts. The U.S. made the initial contribution to the Global Fund to Fight AIDS, Tuberculosis and Malaria, and remains its largest donor today. The U.S. government is also the largest donor country to the Global Alliance for Vaccines and Immunization (GAVI). And, in addition to providing financing to these multilateral mechanisms, the U.S. government is active on their boards, helping to guide the long-term strategic vision of these organizations.

## New Directions Forward

On May 5, 2009, President Barack Obama announced the Global Health Initiative (GHI), the centerpiece of global health for his administration. This is a six-year, $63 billion initiative—a commitment to address some of the most serious health problems facing the world. GHI maintains U.S. funding and strong commitment to existing programs, including the fight against HIV/AIDS, but will also address broader health challenges, including maternal and child health, family planning, infectious diseases, and neglected tropical diseases. In addition, GHI will provide a specific new focus on strengthening health systems and promoting integration and coordination. By connecting US health programs, GHI will allow us to continue our achievements with countries around specific health issues, but also help us to identify and fill gaps in care, services, and systems. Through these efforts, we can ensure that the people we work to support can get coordinated, integrated care for their broader health needs.

GHI identifies core principles that will guide its actions. These include implementing a woman- and girl-centered approach; developing country-led programs; forging deeper partnerships with other global health stakeholders; strengthening health systems; ensuring that U.S. government agencies collaborate for maximum impact; improving metrics, monitoring, and evaluation methods to track results; and ensuring that research, development, and innovation are central to GHI efforts.

GHI is a diplomatic and development undertaking. Its integrated approach will work to improve health systems through focused efforts on major diseases and on maternal and child health issues, while at the same time ensuring that best practices drive funding investments. An important diplomatic goal is to ensure that our partners around the world are well-informed about GHI. Providing this information and promoting understanding about the approach will help identify opportunities to leverage investments or to partner on policy initiatives.

## Emerging Health Challenges

The current influenza A (H1N1) pandemic, when viewed through the global health diplomacy lens, offers additional insights on the linkages between diplomacy and pandemic preparedness. Years before the current H1N1 pandemic, the Department of State began addressing pandemic preparedness, working with international and interagency partners. This included high-level U.S. government participation in ministerial conferences dealing with avian pandemic concerns. In 2005, the U.S. launched the International Partnership for Avian and Pandemic Influenza with the mission of raising awareness and addressing unmet needs. As the H1N1 pandemic unfolded in September 2009, President Obama announced that the U.S. would make as much as 10% of its H1N1 vaccine supply available to other countries through the World Health Organization. The Department of State was involved in the planning process for this initiative and has been at the forefront of working with other nations, which are also donating funding and vaccine. As we look back at the H1N1 response effort, we see the value of diplomatic engagement before, during, and after an acute situation.

## Global Health Diplomacy

Experience in addressing HIV/AIDS and H1N1 shows clearly that addressing global health challenges with diplomacy and development strategies is essential. Secretary Hillary Clinton has stated that one of her policy approaches and a personal priority is: “to elevate and integrate development as a core pillar of American power. We advance our security, our prosperity, and our values by improving the material conditions of people's lives around the world. These efforts also lay the groundwork for greater global cooperation, by building the capacity of new partners and tackling shared problems from the ground up” [1]. The U.S. government works to improve public health and health systems around the world as a humanitarian and development imperative, and as a critical national security requirement. Better global health promotes stability and growth, which can deter the spread of extremism, ease pressure for migration, reduce the need for humanitarian and development assistance, and create opportunities for stronger political alliances and economic relations. Health diplomacy is an element of foreign policy that enhances US and global security and builds strong partnerships.

A second message taken from HIV/AIDS and H1N1 experiences is the importance of partnerships. We must create and sustain partnerships to ensure progress. These are partnerships that cover the globe, engaging health and foreign affairs ministries, bilateral and multilateral organizations, the public and private sectors, and research and health care communities. Our global health work is moved forward through research efforts. Research on disease mechanisms, vaccine development, and program implementation are key to advances in both medical care and program effectiveness. President Obama has spoken clearly on the importance of science and data-based policymaking. In his speech at the National Academy of Sciences on April 27, 2009, [2] he said, “Science is more essential for our prosperity, our security, our health, our environment, and our quality of life than it has ever been before.” He also stated that “[w]e also need to work with our friends around the world. Science, technology and innovation proceed more rapidly and more cost-effectively when insights, costs and risks are shared; and so many of the challenges that science and technology will help us meet are global in character.”

The U.S. Department of State advances global health interests every day. We work closely with the U.S. Agency for International Development, the Centers for Disease Control and Prevention, the National Institutes of Health, and other U.S. federal agencies to understand health priorities and articulate them in diplomatic settings. Staff members move between departments and joint efforts are apparent everywhere. This interplay allows us to have state-of-the-art information and to reflect it to all interested parties. Most importantly, Secretary Clinton is a champion of improving health conditions around the world. Recognizing that foreign policy has evolved to deal with the world we live in, Secretary Clinton has stated, “In approaching our foreign policy priorities we have to deal with the urgent, the important and the long-term all at once” [1]. Today we face a range of issues that confront our nation and the world and that must be addressed to ensure stability and security. Global health is such an issue.
